# DNA microarray profiling of genes differentially regulated by the histone deacetylase inhibitors vorinostat and LBH589 in colon cancer cell lines

**DOI:** 10.1186/1755-8794-2-67

**Published:** 2009-11-30

**Authors:** Melissa J LaBonte, Peter M Wilson, William Fazzone, Susan Groshen, Heinz-Josef Lenz, Robert D Ladner

**Affiliations:** 1 Department of Pathology Norris Comprehensive Cancer Center, Keck School of Medicine, University of Southern California, Los Angeles, CA, USA; 2 Department of Biostatistics Core Norris Comprehensive Cancer Center, Keck School of Medicine, University of Southern California, Los Angeles, CA, USA; 3 Division of Medical Oncology, Norris Comprehensive Cancer Center, Keck School of Medicine, University of Southern California, Los Angeles, CA, USA

## Abstract

**Background:**

Despite the significant progress made in colon cancer chemotherapy, advanced disease remains largely incurable and novel efficacious chemotherapies are urgently needed. Histone deacetylase inhibitors (HDACi) represent a novel class of agents which have demonstrated promising preclinical activity and are undergoing clinical evaluation in colon cancer. The goal of this study was to identify genes in colon cancer cells that are differentially regulated by two clinically advanced hydroxamic acid HDACi, vorinostat and LBH589 to provide rationale for novel drug combination partners and identify a core set of HDACi-regulated genes.

**Methods:**

HCT116 and HT29 colon cancer cells were treated with LBH589 or vorinostat and growth inhibition, acetylation status and apoptosis were analyzed in response to treatment using MTS, Western blotting and flow cytometric analyses. In addition, gene expression was analyzed using the Illumina Human-6 V2 BeadChip array and Ingenuity^® ^Pathway Analysis.

**Results:**

Treatment with either vorinostat or LBH589 rapidly induced histone acetylation, cell cycle arrest and inhibited the growth of both HCT116 and HT29 cells. Bioinformatic analysis of the microarray profiling revealed significant similarity in the genes altered in expression following treatment with the two HDACi tested within each cell line. However, analysis of genes that were altered in expression in the HCT116 and HT29 cells revealed cell-line-specific responses to HDACi treatment. In addition a core cassette of 11 genes modulated by both vorinostat and LBH589 were identified in both colon cancer cell lines analyzed.

**Conclusion:**

This study identified HDACi-induced alterations in critical genes involved in nucleotide metabolism, angiogenesis, mitosis and cell survival which may represent potential intervention points for novel therapeutic combinations in colon cancer. This information will assist in the identification of novel pathways and targets that are modulated by HDACi, providing much-needed information on HDACi mechanism of action and providing rationale for novel drug combination partners. We identified a core signature of 11 genes which were modulated by both vorinostat and LBH589 in a similar manner in both cell lines. These core genes will assist in the development and validation of a common gene set which may represent a molecular signature of HDAC inhibition in colon cancer.

## Background

Within the cellular microenvironment, regulation of gene expression can occur post-transcriptionally through modification of histones and non-histone proteins by acetylation, phosphorylation, methylation, ubiquitination and sumoylation. Two distinct families of enzymes, histone acetyltransferases (HAT) and histone deacetylases (HDAC), work in concert by performing opposing functions to maintain a tightly regulated pattern of acetylation homeostasis. HDACs are zinc-dependent hydrolases which can be classified into 4 different families (class I, IIa, IIb, and IV) that are involved in the remodeling of chromatin by deacetylation of specific lysine residues on histone tails [1,2]. The action of HDACs occurs through formation of large multi-protein complexes with co-activating, co-repressing, and chromatin-remodeling proteins.

It has further been demonstrated that the actions of HDACs and the resultant deacetylation of specific lysine residues is not limited to histones, but occurs on non-histone proteins such as α-tubulin, Hsp90, gluccocorticoid receptors, DNA methyltransferase 1 (DNMT 1) and multiple transcription factors (p53, E2F, GATA1, TFIIE and TFIIF) [3-5]. As such, the role of HDACs in the regulation of cellular processes is more complex than first thought, extending far beyond regulating gene expression and involving active roles in cell-cycle-related processes [6-8]. It is therefore not surprising that dysregulation of HDAC and HAT activity has been identified and reported to contribute to the progression of a number of cancers including leukemia, lymphoma, gastric, prostate, breast and colon [9-13].

Multiple HDAC inhibitors (HDACi) have been developed to date and their administration results in the acetylation of both histone and non-histone proteins, leading to the modulation of between 2 and 10% of expressed genes [14]. The classes of compounds identified as HDACi include: short-chain fatty acids (such as valproic acid), hydroxamic acids (such as TSA, PXD101, LBH589 and vorinostat), cyclic tetrapeptides (such as depsipeptide, FK228) and benzamides (such as MS-275) [15]. Mechanistically, HDACi have been shown to induce G1 and G2/M cell cycle arrest, promote differentiation, induction of apoptotic signaling cascades, mitotic failure, polyploidy and increased generation of reactive oxygen species [16-18]. The hydroxamic acid-based HDACis, vorinostat (SAHA, Merck) [19,20] and LBH589 (panobinostat, Novartis) [21] are pan-inhibitors of class I and II HDACs that have demonstrated potent cytotoxicity *in vitro *against a variety of solid tumor cell lines. Vorinostat is currently FDA-approved for the treatment of cutaneous T-cell lymphoma (CTCL) and is currently in clinical investigation for mesothelioma, non-small cell lung cancer and colon cancer. LBH589 is also under extensive clinical investigation in CTCL and a variety of solid tumors.

Colorectal cancer is the third most commonly diagnosed cancer in both men and women in the United States with a predicted 147,000 new cases in 2009 [22]. Although chemotherapy response rates and patient overall survival rates have improved in recent years [23,24], effective colon cancer treatment is hindered by the high occurrence of drug resistance, subsequent treatment failure and patient mortality, resulting in a critical need to identify and exploit novel therapeutic targets and drug combinations to improve clinical efficacy. HDACi have demonstrated potent activity against colon cancer cell lines *in vitro *and in xenograft models [15,25,26] with little or no cytotoxicity reported against normal cells and clinical evaluations thus far have demonstrated favorable toxicity profiles [27,28].

Several studies to date have demonstrated that HDACi induce alterations in the expression of multiple drug targets and/or metabolic pathways that are critical molecular determinants for cancer therapeutics. Importantly combination treatment with additional agents targeting these modulated pathways has resulted in synergistic growth inhibitory effects on cancer cells *in vitro *and *in vivo*. It has been recently reported that HDACi synergize with 5-FU *in vitro *and *in vivo *in colon cancer cell line models through HDACi-induced downregulation of the 5-FU target enzyme thymidylate synthase (TS), providing a mechanistic basis for the drug synergy [25,29]. The HDACi vorinostat is also reported to acetylate and markedly reduce the chaperone activity of HSP90 in T-cell lymphoma models resulting in a synergistic interaction with the HSP90 inhibitor bortezomib [30]. This combination was subsequently extended to colon cancer cell lines with similar synergistic anti-proliferative effects [31]. In addition, the HDACi vorinostat was demonstrated to induce tumor cell-selective expression of the TRAIL death receptors 4 and 5 sensitizing breast cancer xenografts to the effects of a TRAIL-agonistic antibody [32], an observation which is currently being clinically evaluated in lymphoma patients. More recently, HDACi were also reported to enhance the apoptotic effects of EGFR inhibitors in lung cancer models [33,34] and clinical evaluation of this is ongoing. Therefore, the identification of novel genes modulated by HDACi in colon cancer cells may provide pathway-driven rationale for novel and urgently needed efficacious drug combinations.

This study was designed to determine the effects of two clinically relevant HDACi, vorinostat and LBH589 on the growth characteristics of two cytogenetically distinct colon cancer cell line models HCT116 and HT29. In addition, HDACi-induced alterations in global gene expression were analyzed using the Illumina Human-6 V2 BeachChip arrays and Ingenuity^® ^Pathway Analysis.

## Methods

### Compounds and Reagents

LBH589 was provided by Novartis Pharmaceuticals (East Hanover, NJ). Vorinostat was provided by Merck and Co., Inc. (Whitehouse Station, NJ) and the National Cancer Institute (Bethesda, MD). CellTiter^96 ^AQueous MTS reagent was purchased from Promega (Madison, WI).

### Cell Lines

HCT116 colon cancer cells were a generous gift of Prof. Bert Vogelstein (Johns Hopkins University, Balitmore, MD) and HT29 colon cancer cells were purchased from ATCC (Manassas, VA). HCT116 and HT29 cell lines were maintained in McCoy's 5A medium, supplemented with 10% fetal bovine serum (Lonza, East Rutherford, NJ), penicillin/streptomycin and sodium pyruvate (Invitrogen, Carlsbad, CA). Cells were maintained in a humidified Hepa Class100 Incubator (Thermo, Waltham, MA) at 37°C and 5% CO_2_. Cell lines were routinely screened for mycoplasma using the MycoALERT Detection kit (Lonza).

### Growth Inhibition Assay

Cells were seeded in 96 well plates at 3 × 10^3 ^cells/well in 100 μl of growth media and treated with the indicated concentrations of drug for 72 h at 37°C with 5% CO_2_. MTS (3-(4,5-dimethylthiazol-2-yl)-5-(3-carboxymethoxyphenyl)-2-(4-sulfophenyl)-2H- tetrazolium), assay (Promega) was performed as previously described [35]. Growth inhibition was measured by comparing A490 of drug-treated cells to that of untreated controls set at 100%. The IC_50 _value was calculated from sigmoidal dose-response curves using Prism 5.0 (GraphPad, San Diego, CA). Statistical significance of IC_50 _values between cell lines was evaluated by ANOVA using SAS 9.3.1 statistical software (Cary, NY).

### Flow Cytometric/Sub-G1 Analysis

Cells were seeded at 2.5 × 10^5 ^cells/well in 6-well plates. Duplicate wells were treated with the indicated concentration of drug for 24 h and harvested as previously described [25]. Cells were then analyzed using a Coulter^® ^EPICS^® ^ELITE flow cytometer (Beckman Coulter, Fullerton, CA) equipped with a 15 mW Argon laser (excitation beam 488 nm). Viable cells were gated on a dot plot display of forward scatter versus side scatter to eliminate cell doublets. Cell cycle populations were quantified using histogram analysis software (Expo32, Beckman Coulter). Cells with DNA content <1 were considered apoptotic.

### Western Blotting

Following treatment with indicated concentrations of drug for specified time points, Western blot was performed as described previously [36]. Acetylated histones were detected using anti-acetyl-H3 and anti-acetyl-H4 rabbit antibodies (Upstate). Monoclonal anti-Poly (ADP-Ribose) Polymerase (PARP) was obtained from Cell Signaling (Danvers, MA). Secondary antibodies goat-anti-mouse HRP or goat-anti-rabbit HRP were purchased from Santa Cruz Biotechnology (Santa Cruz, CA). Anti-β-actin was purchased from Sigma (St. Louis, MO) and used to control for loading.

### Microarray Drug Treatments and RNA Isolation

HCT116 and HT29 colon cancer cells were seeded at 7 × 10^6 ^cells/10 cm plate and treated with either 50 nM LBH589 or 2 μM vorinostat for 24 h at 37°C and 5% CO_2_. All treatments were conducted in triplicate and fresh medium was added to untreated control cells. Following the 24 h incubation, cells were harvested and RNA was isolated using the RNeasy^® ^Mini Kit (Qiagen, Valencia, CA) according to the manufacturer's protocol. RNA was subjected to lithium chloride precipitation to remove any possible genomic DNA contamination. The integrity of the RNA was analyzed by spectrophotometry and capillary electrophoresis.

### Microarray Expression Profiling

Microarray expression profiling was performed by the USC/Norris Cancer Center Genomics Core Facility (Los Angeles, CA). The RNA was amplified into cRNA and biotinylated by *in vitro *transcription using the Illumina^® ^TotalPrep RNA Amplification Kit (Ambion, Applied Biosystems, Foster City, CA) according to the manufacturer's protocol. Biotinylated cRNAs were purified, fragmented, and subsequently hybridized to an Illumina Human-6 V2 BeadChip (Illumina, San Diego, CA).

### Data normalization and statistical analysis

Microarray statistical analysis was performed with the assistance of Asuragen Inc., (Austin, TX). The background subtraction, expression summary, normalization, and log base 2 transformation of gene signals were carried out using Quantile Normalization [37]. For statistical analysis, one-way ANOVA was used for multiple group comparison across all samples in the experiment, followed by multiple testing correction to determine the false discovery rate (FDR; Benjamini and Hochberg method [38]). Genes with a FDR-adjusted *p-*value of < 0.05 were considered statistically significant and termed differentially expressed genes (DEGs). Pair-wise comparisons were then performed for all DEGs. For each pair of treatments, a two-sample *t*-test was carried out for every gene, followed by multiple testing correction to determine FDR. The resulting list of genes and associated *p-*values were graphically represented by hierarchical clustering, Venn analysis, principal components analysis (not shown), and volcano plots (not shown).

### Ingenuity^® ^Pathway Analysis (IPA)

In the HCT116 and HT29 cancer cell lines, a total of 3043 and 2232 differentially expressed genes respectively that had FDR-adjusted (*p *< 0.05) were used for the pathway analysis. Gene reference accession numbers were imported into the Ingenuity^® ^Pathway Analysis (IPA) software (Ingenuity^® ^Systems, http://www.ingenuity.com, Mountain View, CA). In the HCT116 and HT29 cancer cell lines, 2289 and 1679 of these genes respectively were mapped to the Ingenuity database. Up- and down-regulated genes were both included as a defined parameter for the core analysis. Genes mapped to genetic networks, were then ranked by a score that defines the probability that a collection of genes equal to or greater than the number in a network can be achieved by chance alone. According to IPA, a score of 3 indicates that there is a 1/1000 chance that the focus genes are in a network due to random chance, and therefore, scores of >3 have a 99.9% confidence of not being generated by random chance alone. This score was used as the cut-off for identifying gene networks that were significantly affected by the HDACi, LBH589 and vorinostat. In a similar way, DEGs were mapped to canonical pathways and tested by the Fishers Exact Test *p*-value. Canonical pathways were represented as a histogram of pathway vs. -log(*p*-value). In addition, for canonical pathways a ratio value was calculated as the number of molecules in a given pathway that meet the cut criteria, divided by the total number of molecules that make up that pathway.

### Quantitative real-time PCR

The abundance of selected transcripts, which had been previously identified by microarray expression profiling at 24 h, was re-evaluated by qPCR at 6, 12, and 24 h. The total RNA was isolated from HCT116 and HT29 colon cancer cells with TRIzol reagent (Invitrogen). RNA (0.5 μg) was reverse transcribed to cDNA using the Promega Reverse Transcription System according to manufacturers instructions and analyzed using an Applied Biosystems 7500 PCR Detection System (Applied Biosystems Inc.). All reactions were performed in triplicate in a final volume of 25 μl. All amplifications were primed by pairs of chemically synthesized 18- to 24-mer olignucleotides designed using freely available primer design software (Primer-BLAST, NCBI) to generate target amplicons of 100-200 bp. Reaction conditions were as follows: Activation at 95°C for 10 min and 40 cycles of denaturation at 95°C for 15 s, annealing at 55°C for 35 s, and extension at 72°C for 45 s. Melt curve analysis of all samples was routinely performed to ascertain that only the expected products had been generated. All primers utilized displayed PCR efficiencies of >90%. Target genes were normalized to GAPDH and quantified using the comparative *C*_T _method described by Livak *et al*. [39] and as used previously [36]. Histograms and statistical analyses (2-tailed unpaired *t*-test) were performed with Prism 5.0 (GraphPad Software).

## Results

### Vorinostat and LBH589 inhibit the growth of colon cancer cells

**Figure 1:**
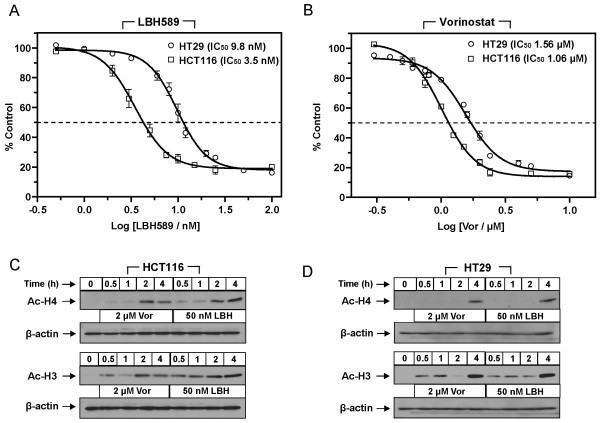
***In vitro *characterization of HDACi, LBH589 and vorinostat, in HCT116 and HT29 colon cancer cells**. HCT116 and HT29 colon cancer cells were exposed to increasing concentrations of either **(A) **LBH589 or **(B) **vorinostat alone for 72 h and subsequent growth inhibition was measured by MTS assay (Promega). Values are presented as percent control, calculated from the growth inhibition induced by a given concentration of drug compared to the untreated control. Values are averages of 3 independent experiments ± SEM. The IC_50(72 h) _values were calculated from the sigmoidal dose-response curves in Prism 5.0 (GraphPad). **(C-D) **Western blot analysis of acetyl-H3 and acetyl-H4 in **(C) **HCT116 and **(D) **HT29 cells treated with 2 μM vorinostat (Vor) or 50 nM LBH589 for 0.5, 1, 2 and 4 h. β-actin was used to control for loading.

The HCT116 and HT29 cell lines were originally derived from human colon adenocarcinomas, and were selected in this study based on marked differences in their cytogenetics. Specifically, these cell lines differ in a number of key genes which have been reported to determine response to chemotherapeutics including the presence of mutant p53 in HT29 cells and activating k-ras and β-catenin mutations in the HCT116 cells. In addition, HCT116 cells display a near-diploid karyotype while HT29 cells exhibit hyper triploidy. These cell lines were initially analyzed to determine the effects of vorinostat and LBH589 on cellular proliferation. Cells were exposed to increasing concentrations of each drug for 72 h and subsequently analyzed by MTS assay. The IC_50(72 h) _values for LBH589 in the HCT116 and HT29 colon cancer cells were in the low nanomolar range at 3.49 nM (95% CI 3.1 - 3.9 nM) and 9.8 nM (95% CI 8.7 - 10.9 μM) respectively (Figure 1A). The IC_50(72 h) _values for vorinostat in the HCT116 and HT29 cells were in the low micromolar range at 1.06 μM (95% CI 0.94 - 1.1 μM) and 1.56 μM (95% CI 1.45 - 1.67 μM) respectively (Figure 1B). The HCT116 cells demonstrated a >2-fold increase in sensitivity to LBH589 (*p *= 0.0019) and a 1.5-fold increase in sensitivity to vorinostat (*p *= 0.027) over the HT29 cells.

### HDACi treatment rapidly induces histone acetylation

Inhibition of HDACs results in disruption of cellular acetylation homeostasis and can induce hyper-acetylation of both histone and non-histone proteins. In order to examine this effect in our colon cancer cell line models, we treated cells with either 2 μM vorinostat or 50 nM LBH589 and analyzed the acetylation status of selected histone proteins. As histone acetylation is reported to be a rapid event following HDACi treatment we analyzed the expression of acetyl-H3 (Ac-H3) and acetyl-H4 (Ac-H4) from 0.5 to 4 h post-treatment. In HCT116 cells, treatment with 2 μM vorinostat resulted in significant Ac-H4 at 2 h post-treatment, however 50 nM LBH589 induced modest but detectable Ac-H4 as early as 0.5 and 1 h post-treatment which increased significantly at 2 and 4 h (Figure 1C and 1D). Interestingly, Ac-H3 was detected as early as 0.5 h post-treatment with both HDACi and increased in a time-dependent manner. In HT29 cells, an increase in Ac-H4 was not detectable following treatment with both HDACi until 4 h post-treatment (Figure 1D). In contrast, Ac-H3 was detected at low levels as early as 0.5 h post-treatment with levels remaining consistent until 4 h post-treatment where a marked increase in Ac-H3 was observed. These results demonstrate that HDACi treatment has detectable and measurable effects on histone acetylation in colon cancer cells within 30 minutes of drug treatment.

### HDACi-induce cell cycle arrest and apoptosis

**Figure 2:**
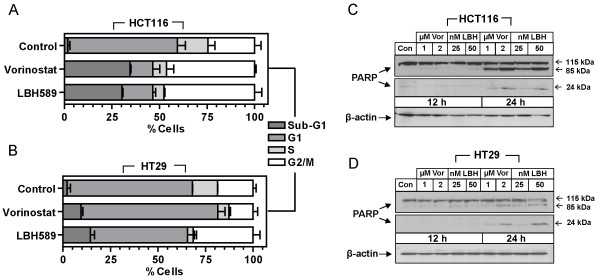
**Cell cycle and apoptotic anlaysis of HDACi-treated colon cancer cells**. Flow cytometric analysis of **(A) **HCT116 and **(B) **HT29 cells treated with 2 μM vorinostat (Vor) or 50 nM LBH589. Histogram bars represent mean ± SEM. **(C-D) **Western blot analysis of poly (ADP-ribose) polymerase (PARP) cleavage as a measure of the induction of apoptosis in HCT116 and HT29 cells treated with 1 and 2 μM vorinostat or 25 and 50 nM LBH589 for 12 and 24 h. β-actin was used to control for loading.

HDACi are reported to rapidly induce cell cycle arrest and induce tumor cell-selective apoptosis. To investigate this, flow cytometry was subsequently utilized to examine the effects of HDACi treatment on cell cycle distribution in HCT116 and HT29 colon cancer cells. Each cell line was treated with 50 nM LBH589 and 2 μM vorinostat (concentrations which were shown to induce similar patterns of histone acetylation) for 24 h and DNA content was subsequently analyzed by propidium iodide staining. The HCT116 colon cancer cells treated with either HDACi, LBH589 or vorinostat, displayed a significant G2/M arrest accompanied by a sharp reduction of cells in G1. Interestingly, cells with subdiploid DNA content (Sub-G1), indicative of cell death, increased from 2% in untreated controls to 30.2 and 34.4% following treatment with LBH589 and vorinostat respectively (Figure 2A). In HT29 cells, treatment with vorinostat resulted in an accumulation of cells arresting in G1 accompanied by a reduction of cells in both G2 and S. Interestingly, LBH589 induced a G2 arrest with a reduction of cells in G1 and S phases (Figure 2B). Despite displaying a similar IC_50(72 h) _value for vorinostat to that of the HCT116 cells, HT29 cells showed only a modest increase in cell death from 2% to 9.5% following treatment with vorinostat. Similarly, despite the concentration of LBH589 being in excess of the IC_50(72 h) _value for HT29 cells, cell death increased modestly from 2% to 14.4% (Figure 2B). These data suggest that while both cell lines display similar sensitivity to the growth inhibitory effects of HDACi, the HT29 cells are significantly more resistant to the onset of HDACi-induced apoptosis in this time-frame. To confirm these differential levels of HDACi-induced apoptosis, HCT116 and HT29 cells were analyzed for the cleavage of poly (ADP-ribose) polymerase (PARP) (a hallmark of apoptosis) from its native 115 kDa to the 89 kDa subunit by Western blot. Compared to vehicle-treated cells, HCT116 cells displayed strong dose-dependent cleavage of PARP at 24 h post treatment evidenced in particular by the strong immunoreactivity of the 85 kDa subunit when compared to the full length PARP (Figure 2C). Twenty-four h post-treatment, PARP cleavage was detected at low levels in HT29 cells in a dose-dependent manner as evidenced by the appearance of the cleaved subunits (Figure 2D). These results support the flow cytometric analysis whereby HCT116 are significantly more susceptible to rapid HDACi-induced apoptosis than the HT29 cells.

### Microarray profiling in HDACi treated colon cancer cells

**Figure 3:**
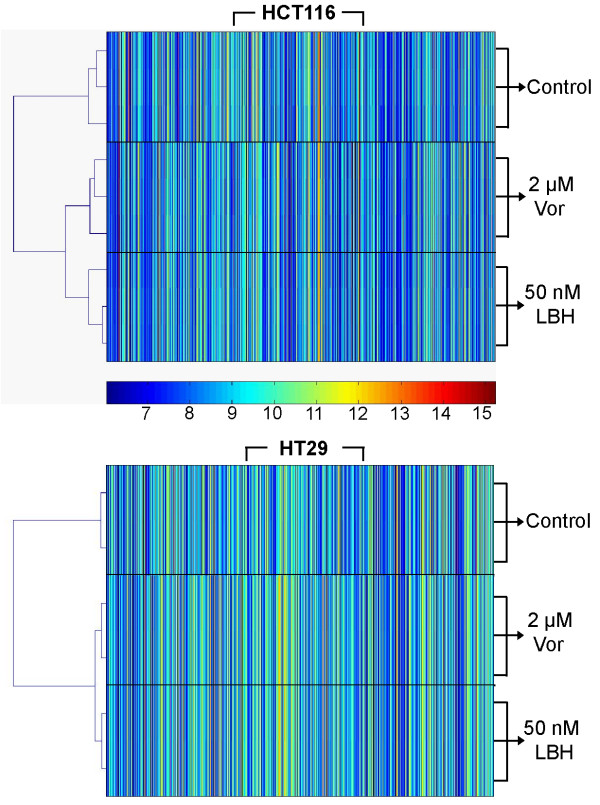
**Hierarchical cluster analysis of HDACi-treated HCT116 and HT29 colon cancer cells**. Cells were treated with 2 μM vorinostat or 50 nM LBH589 for 24 h and gene expression was analyzed using the Illumina Human-6 V2 BeadChip array. Hierarchical cluster heat map and tree was generated from HDACi-induced changes in gene expression (1-way ANOVA, p < 0.05).

To identify the molecular events which occur in response to HDAC inhibition in colon cancer cells, we treated both HCT116 and HT29 colon cancer cells with the clinically relevant concentrations of 50 nM LBH589 or 2 μM vorinostat for 24 h, isolated mRNA and subsequently analyzed gene expression using the Illumina Human-6 V2 BeadChip array platform as outlined in the methods section. Genes with a FDR-adjusted *p-*value of < 0.05 were considered differentially expressed genes (DEGs) relative to vehicle treated controls. The heat maps generated from the microarray analysis in HCT116 and HT29 cells treated with HDACi were subject to hierarchical clustering analysis. The heat map demonstrates that both vorinostat and LBH589 segregated independently from the vehicle-treated controls in both cell lines. However, the cluster tree generated also demonstrates that while vorinostat and LBH589 segregate from the vehicle-treated controls, they demonstrate very similar clustering patterns indicating that they induce similar transcriptional response within each cell line (Figure 3).

### Differentially expressed genes in response to HDACi treatment

**Figure 4:**
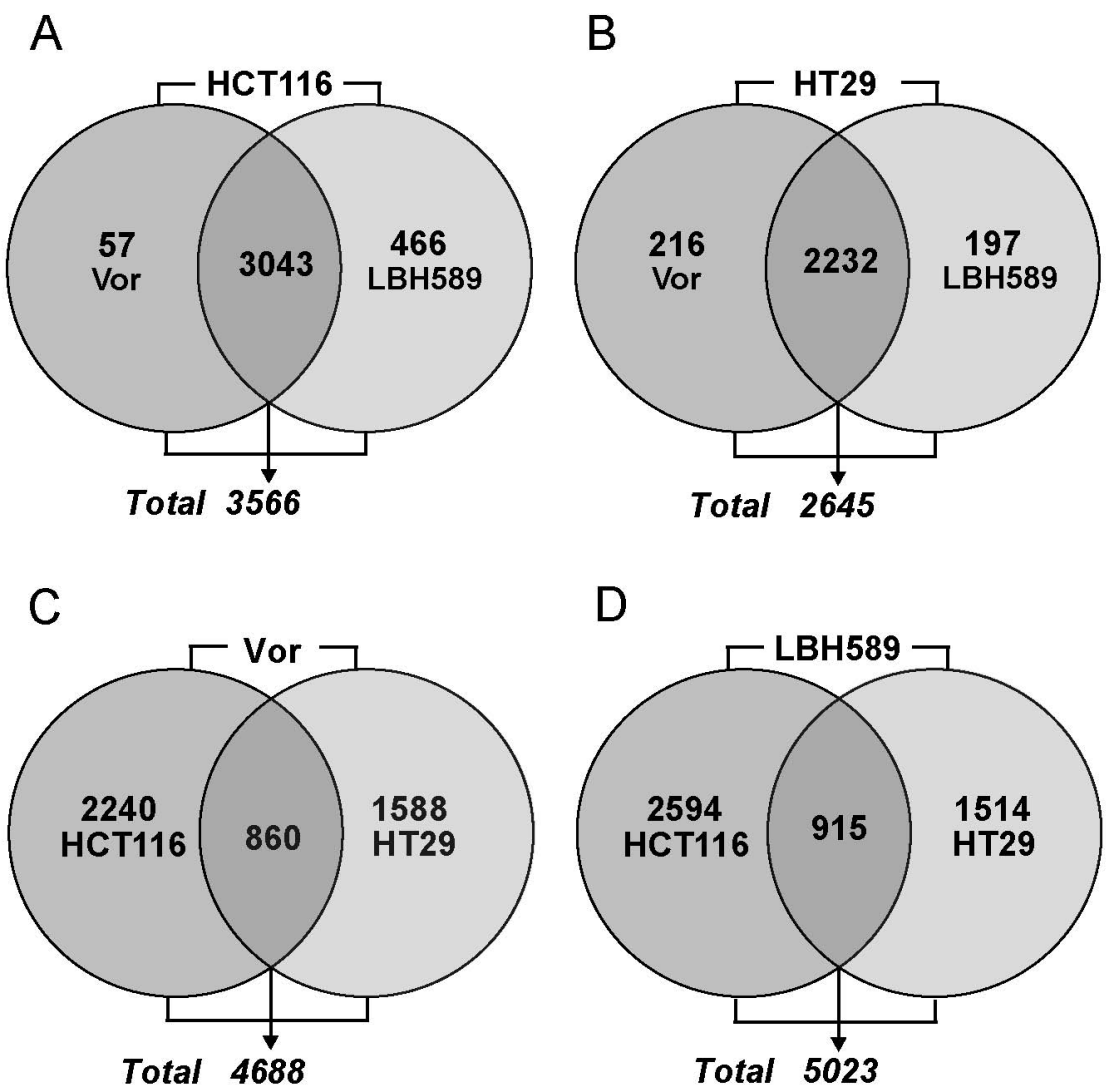
**Venn analysis of differentially expressed genes in vorinostat and LBH589-treated HCT116 and HT29 colon cancer cells**. HCT116 and HT29 cells were treated with either 2 μM vorinostat or 50 nM LBH589 for 24 h and gene expression analyzed on the Illumina Human-6 V2 BeadChip array. Genes with an FDR-adjusted *p*-value of < 0.05 were considered differentially expressed and subjected to Venn analysis. Venn analysis was first performed by analyzing cell-line-specific alterations in each individual cell line; **(A) **HCT116 cells treated with vorinostat or LBH589. **(B) **HT29 cells treated with vorinostat or LBH589. Subsequent Venn analysis demonstrates the drug-specific alterations induced by **(C) **vorinostat (Vor) and **(D) **LBH589 in both cell lines. Numbers within each circle represent the total number of genes modulated in that experimental condition, the numbers immediately below each Venn diagram indicate the total number of modulated genes by both experimental conditions in that Venn diagram.

To compare the effects of HDACi treatment relative to vehicle-treated cells, Venn analysis was utilized. In HCT116 cells, a combined total of 3566 genes were modulated by HDACi treatment (both LBH589 or vorinostat) representing approximately 7% of the total gene set analyzed by the array. Within this set, 3100 DEGs were identified following vorinostat treatment of which 57 genes were uniquely modulated by vorinostat treatment as illustrated by the Venn diagram (Figure 4A). Following treatment with LBH589, 3509 DEGs were identified of which 466 genes were uniquely modulated by LBH589 (Figure 4A). This data demonstrates that in HCT116 cells, vorinostat and LBH589 exert similar effects on gene expression with 85% of all DEGs modulated in a consistent manner by both vorinostat and LBH589 treatment.

In HT29 cells, 2645 genes were modulated in total by both HDACi representing approximately 5% of the total gene set analyzed by the array (Figure 4B). Of this total, 2448 genes were modulated by vorinostat of which 216 of these DEGs were unique only to vorinostat treatment (Figure 4B). Following treatment with LBH589, 2429 genes were modulated of which 197 were unique transcriptional responses to LBH589 not observed with vorinostat treatment (Figure 4B). This indicates that there is also significant similarity in the transcriptional changes induced by vorinostat and LBH589 in HT29 cells with 75% of the total DEG set common transcriptional changes in response to either HDACi.

Of the 3100 DEGs modulated by vorinostat treatment in HCT116 cells, 24 were up-regulated and 17 were down-regulated >2-fold. Of the 3509 genes modulated following treatment with LBH589 in HCT116 cells, 92 genes were upregulated and 150 were downregulated >2-fold. The top 15 up- and downregulated genes modulated >2-fold for both vorionostat and LBH589 treatment in HCT116 cells are displayed in Table 1.

**Table 1:** Differentially expressed genes (>2 Fold) in response to HDACi in HCT116 colon cancer cells.

			HCT116	
**Accession #**	**Gene Symbol**	**Gene Name**	**Fold Change**	***P*-Value**
**Induced**			**LBH589**	
NM_001901.2	CTGF	Connective tissue growth factor	6.09	6.9E-04
NM_182908.3	DHRS2	Dehydrogenase/reductase member 2	4.78	1.7E-04
NM_003378.2	VGF	VGF nerve growth factor inducible	4.74	1.0E-04
NM_183376.1	ARRDC4	Arrestin domain containing 4	4.15	6.9E-04
NM_173798.2	ZCCHC12	Zinc finger, CCHC domain containing 12	4.13	1.8E-05
NM_006865.2	LILRA3	Leukocyte immunoglobulin-like receptor, subfamily A, member 3	3.94	7.2E-05
NM_033184.2	KRTAP2-4	Keratin associated protein 2-4	3.61	7.2E-04
NM_000558.3	HBA1	Hemoglobin, alpha 1	3.60	3.2E-04
NM_016352.3	CPA4	Carboxypeptidase A4	3.47	2.7E-04
NM_001554.4	CYR61	Cysteine-rich, angiogenic inducer, 61	3.44	2.7E-05
NM_005319.3	HIST1H1C	Histone 1, H1c	3.37	9.9E-05
NM_138720.1	HIST1H2BD	Histone 1, H2bd	3.35	2.2E-04
NM_031476.1	CRISPLD2	Cysteine-rich secretory protein LCCL domain containing 2	3.26	8.4E-05
NM_139072.2	DNER	Delta-notch-like EGF repeat-containing transmembrane	3.21	1.5E-03
NM_005061.2	RPL3L	Ribosomal protein L3-like	3.08	2.5E-05
**Repressed**				
NM_013233.2	STK39	Serine threonine kinase 39	-2.91	8.2E-04
NM_004091.2	E2F2	E2F transcription factor 2	-2.93	2.4E-04
NM_003302.1	TRIP6	Thyroid hormone receptor interactor 6	-2.99	7.3E-04
NM_005733.2	KIF20A	Kinesin family member 20A	-3.00	2.2E-05
NM_005329.2	HAS3	Hyaluronan synthase 3	-3.02	4.1E-05
NM_145810.1	CDCA7	Cell division cycle associated 7	-3.06	2.4E-03
NM_005434.4	MALL	Mal, T-cell differentiation protein-like	-3.07	2.8E-04
NM_002129.2	HMGB2	High-mobility group box 2	-3.13	3.6E-03
NM_018649.2	H2AFY2	H2A histone family, member Y2	-3.28	8.7E-04
NM_001038.5	SCNN1A	Sodium channel, nonvoltage-gated 1	-3.59	6.6E-04
NM_004217.2	AURKB	Aurora kinase B	-3.61	5.4E-04
NM_001237.3	CCNA2	Cyclin A2	-3.72	2.9E-05
NM_001071.1	TYMS	Thymidylate synthase	-3.88	1.1E-04
NM_181803.1	UBE2C	Ubiquitin-conjugating enzyme E2C	-3.95	2.6E-04
NM_001423.2	EMP1	Epithelial membrane protein 1	-4.17	1.3E-05
				
**Induced**			**Vorinostat**	
NM_001901.2	CTGF	Connective tissue growth factor	4.76	2.0E-05
NM_003378.2	VGF	VGF nerve growth factor inducible	4.35	1.8E-03
NM_182908.3	DHRS2	Dehydrogenase/reductase member 2	4.05	6.2E-04
NM_183376.1	ARRDC4	Arrestin domain containing 4	3.35	4.4E-04
NM_173798.2	ZCCHC12	Zinc finger, CCHC domain containing 12	3.27	1.7E-06
NM_016352.3	CPA4	Carboxypeptidase A4	2.91	1.0E-03
NM_017445.1	H2BFS	H2B histone family, member S	2.86	2.8E-04
NM_138720.1	HIST1H2BD	Histone 1, H2bd	2.85	1.6E-04
NM_033184.2	KRTAP2-4	Keratin associated protein 2-4	2.81	2.6E-03
NM_001554.4	CYR61	Cysteine-rich, angiogenic inducer, 61	2.73	1.3E-05
NM_139072.2	DNER	Delta-notch-like EGF repeat-containing transmembrane	2.62	2.2E-03
NM_005319.3	HIST1H1C	Histone 1, H1c	2.57	5.4E-04
NM_006865.2	LILRA3	Leukocyte immunoglobulin-like receptor, subfamily A, member 3	2.37	1.2E-03
NM_005952.2	MT1X	Metallothionein 1×	2.37	1.2E-03
NM_080593.1	HIST1H2BK	Histone 1, H2bk	2.36	9.2E-06
NM_005950.1	MT1G	Metallothionein 1G	2.33	7.4E-03
**Repressed**				
NM_003998.2	NFKB1	Nuclear factor of kappa light polypeptide gene enhancer in B-cells 1 (p105)	-2.04	6.9E-04
NM_018043.5	TMEM16A	Transmembrane protein 16A	-2.05	1.6E-03
NM_003302.1	TRIP6	Thyroid hormone receptor interactor 6	-2.08	6.6E-03
NM_145810.1	CDCA7	Cell division cycle associated 7	-2.08	2.4E-03
NM_004217.2	AURKB	Aurora kinase B	-2.08	1.7E-03
NM_001235.2	SERPINH1	Serpin peptidase inhibitor, clade H (heat shock protein 47), member 1	-2.11	7.4E-05
NM_001425.2	EMP3	Epithelial membrane protein 3	-2.11	1.0E-03
NM_005329.2	HAS3	Hyaluronan synthase 3	-2.11	2.4E-05
NM_001237.3	CCNA2	Cyclin A2	-2.15	1.5E-03
NM_002129.2	HMGB2	High-mobility group box 2	-2.17	1.3E-03
NM_005434.4	MALL	Mal, T-cell differentiation protein-like	-2.17	4.2E-04
NM_001423.2	EMP1	Epithelial membrane protein 1	-2.23	1.6E-04
NM_181803.1	UBE2C	Ubiquitin-conjugating enzyme E2C	-2.31	3.1E-04
NM_018649.2	H2AFY2	H2A histone family, member Y2	-2.51	2.2E-06
NM_001071.1	TYMS	Thymidylate synthase	-2.74	7.4E-06

Similarly, in HT29 cells, the majority of DEGs were also modulated <2-fold when compared to vehicle-treated controls as was observed in the HCT116 cells. Of the 2448 DEGs modulated by vorinostat treatment in HT29 cells, 138 were up-regulated and 53 were down-regulated >2-fold. Of the 3509 genes modulated following treatment with LBH589 in HT29 cells, 163 genes were upregulated and 54 were downregulated >2-fold. The top 15 up- and downregulated genes for both vorinostat and LBH589 treatment in HT29 cells are displayed in Table 2.

**Table 2:** Differentially expressed genes (>2 Fold) in response to HDACi in HT29 colon cancer cells.

			HT29	
**Accession #**	**Gene Symbol**	**Gene Name**	**Fold Change**	***P*-Value**
**Induced**			**LBH589**	
NM_002305.2	LGALS1	Lectin, galactoside-binding soluble 1	5.22	3.0E-03
NM_003088.2	FSCN1	Fascin homolog 1, actin-bundling protein	5.06	5.6E-03
NM_006262.3	PRPH	Peripherin	4.58	9.8E-04
NM_004223.3	UBE2L6	Ubiquitin-conjugating enzyme E2L 6	4.58	9.7E-04
NM_182908.3	DHRS2	Dehydrogenase/reductase member 2	4.37	5.5E-03
NM_006086.2	TUBB3	Tubulin, beta 3	4.06	1.4E-02
NM_002084.2	GPX3	Glutathione peroxidase 3	4.05	5.2E-03
NM_153247.1	SLC29A4	Solute carrier family 29, member 4	3.92	1.1E-03
NM_003283.3	TNNT1	Troponin T type 1	3.92	1.3E-03
NM_178012.3	TUBB2B	Tubulin, beta 2B	3.92	3.4E-03
NM_001928.2	CFD	Complement factor D (adipsin)	3.87	5.4E-03
NM_006117.2	PECI	Peroxisomal D3, D2-enoyl-CoA isomerase	3.83	6.8E-03
NM_005319.3	HIST1H1C	Histone 1, H1c	3.78	1.3E-03
NM_005952.2	MT1X	Metallothionein 1×	3.66	5.9E-03
NM_017707.2	DDEFL1	Development and differentiation enhancing factor-like 1	3.60	6.6E-04
**Repressed**				
NM_206963.1	RARRES1	Retinoic acid receptor responder 1	-2.41	1.0E-02
NM_001031733.1	CALML4	Calmodulin-like 4	-2.42	1.1E-03
NM_007167.2	ZMYM6	Zinc finger, MYM-type 6	-2.43	8.8E-03
NM_002423.3	MMP7	Matrix metallopeptidase 7	-2.55	7.1E-03
NM_080911.1	UNG	Uracil-DNA glycosylase	-2.58	1.1E-02
NM_145810.1	CDCA7	Cell division cycle associated 7	-2.59	2.0E-03
NM_006169.2	NNMT	Nicotinamide N-methyltransferase	-2.61	4.4E-03
NM_020371.2	AVEN	Apoptosis, caspase activation inhibitor	-2.66	4.3E-04
NM_005375.2	MYB	V-myb myeloblastosis viral oncogene homolog	-2.72	1.2E-03
NM_052813.2	CARD9	Caspase recruitment domain family, member 9	-2.75	1.8E-02
NM_014312.3	VSIG2	V-set and immunoglobulin domain containing 2	-2.81	2.5E-03
NM_020384.2	CLDN2	Claudin 2	-3.03	2.5E-02
NM_020299.3	AKR1B10	Aldo-keto reductase family 1, member B10	-3.20	1.7E-02
NM_007193.3	ANXA10	Annexin A10	-3.69	4.1E-03
NM_001071.1	TYMS	Thymidylate synthase	-3.82	1.1E-03
				
**Induced**			**Vorinostat**	
NM_002305.2	LGALS1	Lectin, galactoside-binding, soluble, 1	5.07	1.6E-03
NM_003088.2	FSCN1	Fascin homolog 1, actin-bundling protein	4.61	2.3E-04
NM_004223.3	UBE2L6	Ubiquitin-conjugating enzyme E2L 6	4.47	1.2E-02
NM_006262.3	PRPH	Peripherin	4.39	5.9E-03
NM_182908.3	DHRS2	Dehydrogenase/reductase member 2	4.21	1.1E-04
NM_006086.2	TUBB3	Tubulin, beta 3	4.08	1.6E-03
NM_002084.2	GPX3	Glutathione peroxidase 3	3.98	2.0E-03
NM_017707.2	DDEFL1	Development and differentiation enhancing factor-like 1	3.74	7.9E-03
NM_001928.2	CFD	Complement factor D	3.72	1.5E-02
NM_003078.3	SMARCD3	SWI/SNF related, matrix associated, actin dependent regulator of chromatin, subfamily d, member 3	3.67	2.0E-02
NM_003283.3	TNNT1	Troponin T type 1	3.67	1.5E-03
NM_006117.2	PECI	Peroxisomal D3, D2-enoyl-CoA isomerase	3.62	3.5E-03
NM_178012.3	TUBB2B	Tubulin, beta 2B	3.59	7.1E-04
NM_015896.2	ZMYND10	Zinc finger, MYND-type containing 10	3.57	8.8E-03
NM_005319.3	HIST1H1C	Histone 1, H1c	3.50	7.7E-03
**Repressed**				
NM_001031733.1	CALML4	Calmodulin-like 4	-2.43	5.0E-03
NM_005752.2	CLEC3A	C-type lectin domain family 3, member A	-2.45	7.5E-04
NM_005375.2	MYB	V-myb myeloblastosis viral oncogene homolog	-2.56	3.5E-03
NM_020371.2	AVEN	Apoptosis, caspase activation inhibitor	-2.60	7.8E-03
NM_014312.3	VSIG2	V-set and immunoglobulin domain containing 2	-2.62	3.5E-04
NM_005117.2	FGF19	Fibroblast growth factor 19	-2.64	9.3E-03
NM_007167.2	ZMYM6	Zinc finger, MYM-type 6	-2.67	1.6E-02
NM_004688.1	NMI	N-myc (and STAT) interactor	-2.69	3.9E-04
NM_052813.2	CARD9	Caspase recruitment domain family, member 9	-2.70	2.8E-04
NM_080911.1	UNG	Uracil-DNA glycosylase	-2.79	1.6E-02
NM_018689.1	KIAA1199	KIAA1199	-2.95	3.2E-04
NM_020384.2	CLDN2	Claudin 2	-3.06	2.3E-02
NM_020299.3	AKR1B10	Aldo-keto reductase family 1, member B10	-3.07	2.8E-05
NM_007193.3	ANXA10	Annexin A10	-3.49	1.3E-02
NM_001071.1	TYMS	Thymidylate synthase	-3.59	8.3E-03
NM_001031733.1	CALML4	Calmodulin-like 4	-2.43	5.0E-03

### Identification of biological pathways modulated by HDACi

**Figure 5:**
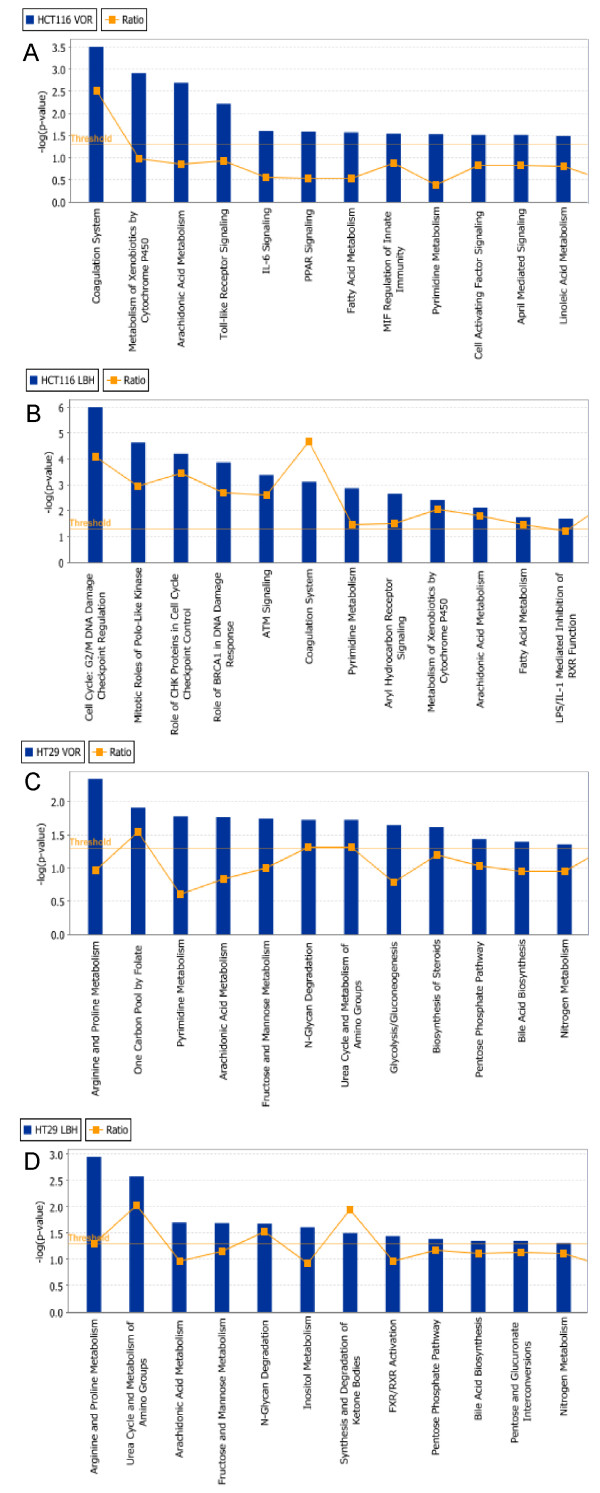
**Top 12 canonical pathways that were significantly modulated by HDACi as identified by Ingenuity^® ^Pathway Analysis (IPA)**. HCT116 colon cancer cells treated for 24 h with **(A) **2 μM vorinostat (Vor) or **(B) **50 nM LBH589 (LBH); HT29 colon cancer cells treated for 24 h with **(C) **2 μM vorinostat (Vor) or **(D) **50 nM LBH589 (LBH). 2289 of the 3043 differentially expressed genes (DEGs) in the HCT116 and 1679 of the 2232 DEGs in the HT29 cancer cell lines mapped to defined genetic networks in IPA. Fisher's exact test was used to calculate a *p*-value determining the probability that the association between the genes in the dataset and the canonical pathway is explained by chance alone. A ratio of the number of genes from the dataset that map to the pathway divided by the total number of molecules in a given pathway that meet the cut criteria, divided by the total number of molecules that make up that pathway is displayed.

We further analyzed the HDACi-DEGs to explore the key biological pathways modulated by HDACi treatment. We performed pathway analysis using Ingenuity^® ^Pathway Analysis (IPA) on the DEGs in both the HCT116 and HT29 cell lines, treated with LBH589 and vorinostat. In the HCT116 cells, 2289 of the 3043 DEGs and 1679 of the 2232 DEGs in the HT29 cells mapped to defined genetic networks in IPA Knowledge Base. Five networks were found to be altered by HDACi in that they possessed significantly more of the identified DEGs present than would be expected by random chance. These networks included cell cycle; DNA replication, recombination and repair; apoptosis; gene expression and cell growth and proliferation. The mapped DEGs were subsequently analyzed for the top 12 canonical biological pathways that demonstrated significance within each dataset. In HCT116 cells, 5 common pathways were modulated by both HDACi; coagulation system, pyrimidine metabolism, metabolism of xenobiotics, arachidonic acid metabolism and fatty acid metabolism (Figure 5A and 5B). In HT29 cells, 7 common pathways were modulated by both HDACi; arginine and proline metabolism; urea cycle and metabolism of amino groups; arachidonic acid metabolism; fructose and mannose metabolism; pentose phosphotate pathway; nitrogen metabolism and bile acid biosynthesis (Figure 5C and 5D).

### Common gene signature of HDAC inhibition in colon cancer cells

One of the key objectives of this study was to identify a cassette of genes which were consistently regulated by both vorinostat and LBH589 in both cell lines examined. Such a cassette of consistently regulated genes may serve as molecular markers for HDACi treatment in colon cancer cells.

From the Venn analysis, it is apparent that there is significant differences in how the HCT116 and HT29 cells respond to HDACi treatment. Specifically, when HCT116 and HT29 cells were treated with vorinostat, a combined total of 4688 DEGs (*p*-value < 0.05) were identified. However, of this combined total of 4688 DEGs, only 860 (18.3%) genes were transcriptional changes common to both cell lines (Figure 4C and 4D). Similarly, in both cell lines a combined total of 5023 DEGs (*p*-value < 0.05) were identified following treatment with LBH589. However, of these 5023 DEGs, only 915 (18.2%) were transcriptional changes common to both cell lines (Figure 4C and 4D). From this overlapping gene list, up- and downregulated genes in the HCT116 and HT29 cells were directly compared using a 1.5-fold cutoff. From this comparative list, we identified a panel of 11 genes, 6 genes that are significantly upregulated and 5 that are downregulated in a consistent manner in both cell lines by treatment with both HDACi (Table 3).

**Table 3:** Summary of changes in gene expression for the core set of HDAC inhibitor regulated genes

			HCT116		HT29	
**Accession #**	**Gene Symbol**	**Gene Name**	Fold Change*		Fold Change*	
**Induced**			**LBH589**	**VOR**	**LBH589**	**VOR**
NM_182908.3	DHRS2	Dehydrogenase/reductase member 2	4.78	4.05	4.37	4.21
NM_183376.1	ARRDC4	Arrestin domain containing 4	4.15	3.35	2.21	2.14
NM_138720.1	HIST1H2BD	Histone 1, H2bd	3.35	2.53	3.44	3.33
NM_005952.2	MT1X	Metallothionein 1X	2.87	2.37	3.66	3.31
NM_005950.1	MT1G	Metallothionein 1G	2.78	2.08	3.30	3.00
NM_015149.2	RGL1	Tal guanine nucleotide dissociation stimulator-like 1	2.52	1.56	3.12	3.03
						
**Repressed**						
NM_001071.1	TYMS	Thymidylate synthase	-3.88	-2.74	-3.82	-3.59
NM_145810.1	CDCA7	Cell division cycle associated 7	-3.06	-2.08	-2.59	-2.42
NM_080911.1	UNG	Uracil-DNA glycosylase	-2.54	-1.55	-2.58	-2.79
NM_003998.2	NFkB1	Nuclear factor of kappa light polypeptide gene enhancer in B-cells 1 (p105)	-2.55	-2.04	-1.65	-1.8
NM_001025242.1	IRAK1	Interleukin-1 receptor-associated kinase 1	-1.97	-1.65	-2.15	-2.08

### Verification of microarray results by quantitative real-time RT-PCR

**Figure 6:**
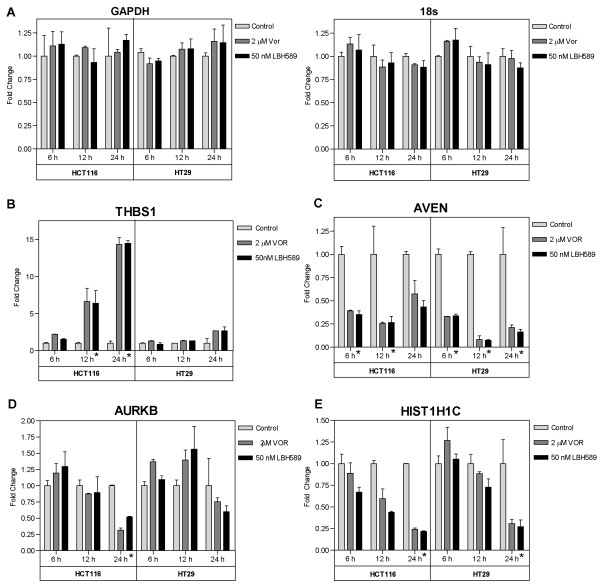
**qPCR validation of house-keeping and cell-line specific HDACi-induced gene expression changes**. HCT116 and HT29 cells were treated with 2 μM vorinostat or 50 nM LBH589 for 6, 12 and 24 h. Total RNA was extracted and qPCR analysis was performed as described in the 'materials and methods' using the primer sets given in Table 4. Histogram bars represent the mean ± SD for two independent RNA isolations analyzed in triplicate. **(A) **Verification of unaffected 18s and GAPDH expression with HDACi treatment. GAPDH was normalized to 18s and 18s was normalized to GAPDH. qPCR validation of the induction of **(B) **THBS-1, **(C) **AVEN **(D) **AURKB **(E) **HIST1H1C. All genes were normalized to GAPDH, * denotes a *p*-value < 0.05 for both HDACi treatment groups when compared to respective time-matched control.

**Figure 7:**
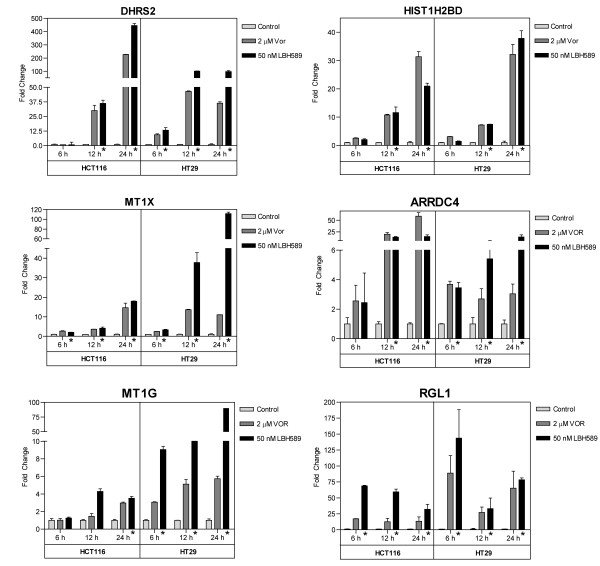
**qPCR time-dependent validation of core HDACi-induced gene expression changes in HCT116 and HT29 cells**. HCT116 and HT29 cells were treated with 2 μM vorinostat (Vor) or 50 nM LBH589 for 6, 12 and 24 h. Total RNA was extracted, reverse transcribed and qPCR analysis was performed as described in the 'materials and methods' using the primer sets given in Table 4. Histogram bars represent the mean ± SD for two independent RNA isolations analyzed in triplicate. All genes were normalized to GAPDH, * denotes *p*-value < 0.05 for both HDACi treatment groups when compared to respective time-matched control.

**Figure 8:**
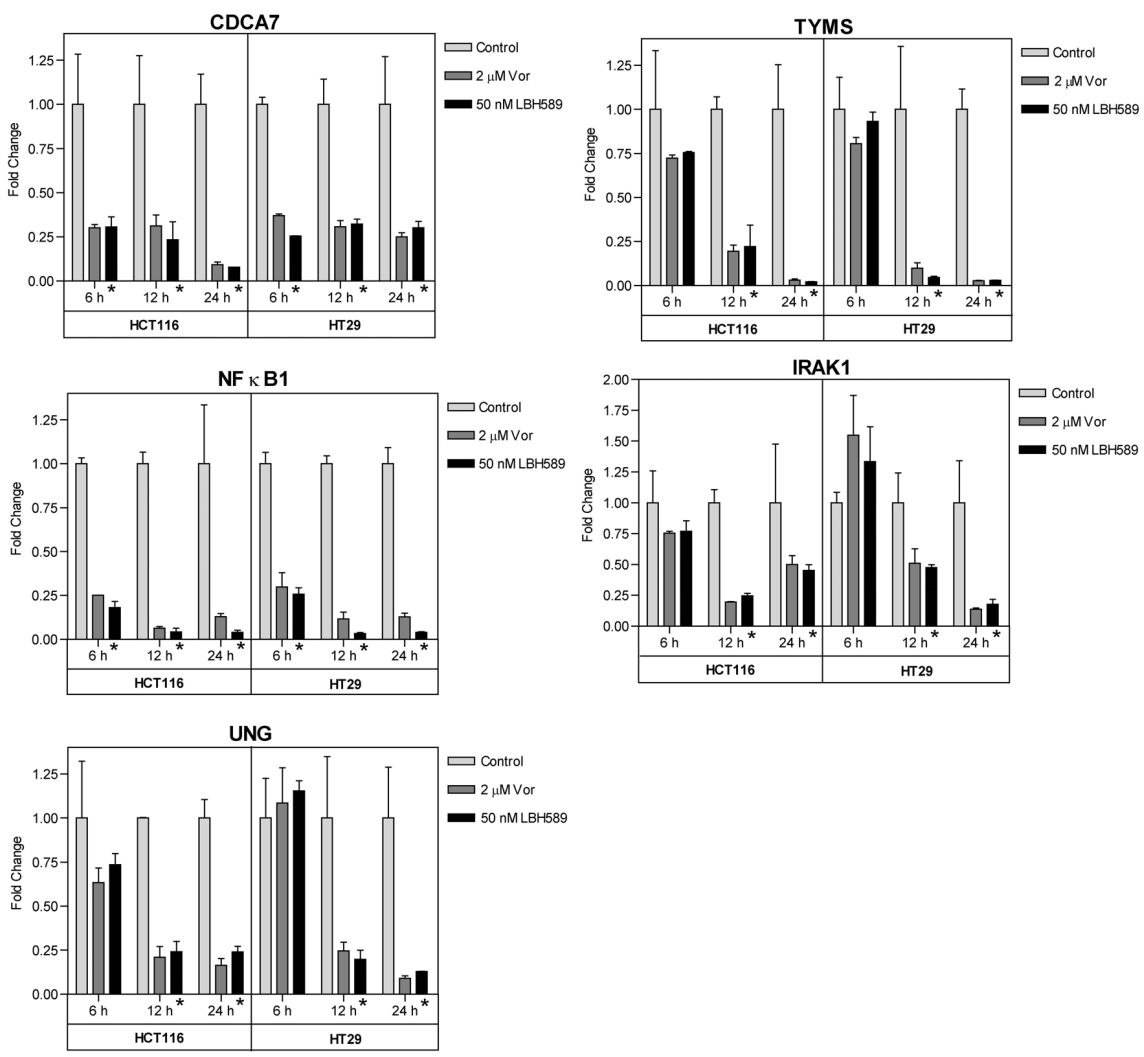
**qPCR time-dependent validation of core HDACi-repressed gene expression changes in HCT116 and HT29 cells**. HCT116 and HT29 cells were treated with 2 μM vorinostat (Vor) or 50 nM LBH589 for 6, 12 and 24 h. Total RNA was extracted, reverse transcribed and qPCR analysis was performed as described in the 'materials and methods' using the primer sets given in Table 4. Histogram bars represent the mean ± SD for two independent RNA isolations analyzed in triplicate. All genes were normalized to GAPDH, * denotes *p*-value < 0.05 for both HDACi treatment groups when compared to respective time-matched control.

In order to assess the robustness of the microarray analysis, quantitative real-time RT-PCR (qPCR) analysis was performed to validate a selected panel of 15 DEGs and 2 non-DEGs [39], using the primer sets given in Table 4. qPCR was performed on cDNA generated using RNA independently isolated from HCT116 and HT29 treated with either 2 μM vorinostat or 50 nM LBH589. Due to the pleotropic effects on gene expression induced by HDACi, we first confirmed that our selected qPCR normalizing gene was not modulated by HDACi treatment in either cell line prior to DEG verification. We selected 2 house-keeping genes, 18s rRNA and GAPDH, whose expression was unchanged in the microarray analysis (non-DEGs) and confirmed using qPCR that these genes retained consistent expression during HDACi treatment (Figure 6A). GAPDH was subsequently used to normalize all qPCR data. To further validate and characterize the DEGs identified by the microarray analysis, we analyzed the time-dependent change in expression of the selected DEGs at 6, 12 and 24 hours post-HDACi treatment when compared to vehicle-treated time-matched controls. The pattern of expression obtained for 14 of the 15 selected DEGs 24 h post-treatment showed consistent directional conformation (up- or downregulation) and cell-line specific modulation between the qPCR and microarray analyses. THBS-1, AVEN and AURKB demonstrated significant cell-line specific changes in expression at 24 h as observed in the microarray analyses (Figure 6B-D). HIST1H1C was initially identified as consistently upregulated by the microarray analysis, but subsequent qPCR analysis indicated this gene to be consistently downregulated with either HDACi in both cell lines (Figure 6E). QPCR validation of our core panel of 11 genes also demonstrated consistent modulation at 24 h with the microarray analysis. In addition, these 11 genes also demonstrated time-dependent changes in expression at 6 and/or 12 h post HDACi treatment (Figure 7 and 8). However, in several instances, the fold-changes obtained by qPCR were significantly higher for several genes than those obtained in the microarray analyses, particularly for the more heavily regulated genes as previously reported [40]. For example, DHRS2 was induced by ~5-fold in both cell lines following HDACi treatment in the microarray analysis. Subsequent qPCR analysis determined the fold-increase in DHRS2 transcripts to be in the order of 36-97-fold in HT29 cells and 226-445-fold in the HCT116 cells (Figure 7). Similarly, thymidylate synthase (TYMS) was down-regulated by HDACi in both cell lines 2.7 - 3.8-fold in the microarray analysis, whereas qPCR determined that HDACi treatment induced a >30-fold downregulation of TYMS 24 h post-treatment in both cell lines (Figure 8).

**Table 4:** qPCR Primer Sequences

Gene				
**Name**	**Accession #**	**Strand**	**Primer Sequence**	**Size (bp)**
18s rRNA	NR_003286.1	F	CGCCGCTAGAGGTGAAATTC	62
		R	TTGGCAAATGCTTTCGCTC	
ARRDC4	NM_183376.2	F	CCGGCCGGTGAAGGCATCAT	140
		R	TCCAACACTGCCCGCACACA	
AURKB	NM_004217.2	F	GACCTAAAGTTCCCCGCTTC	165
		R	GACAGATTGAAGGGCAGAGG	
AVEN	NM_020371.2	F	TGCTCACAGCAGTAAATGCC	155
		R	TGCAAGGAAGGAGGCTAGAA	
CDCA7	NM_031942.4	F	CATGGAAACCTCGTCATCCT	95
		R	TACAGCCTTCCCGAACTGAC	
DHRS2	NM_182908.4	F	GTCCTTCCTGTGCTCTCCAG	169
		R	AGACTCTGCCTCCAGATCCA	
GAPDH	NM_002046.3	F	ATGGGGAAGGTGAAGGTCG	107
		R	GGGTCATTGATGGCAACAATATC	
HIST1H1C	NM_005319.3	F	ACACCGAAGAAAGCGAAGAA	116
		R	AGCCTTAGCAGCACTTTTGG	
HIST1H2BD	NM_138729.1	F	AAGGCCGTCACCAAGTACAC	136
		R	TTTCAGGCAGATGAGACTTCC	
IRAK1	NM_0001569.3	F	GCTGGCCCTGTACGAGGAT	129
		R	ACACATCAGCTCTGAAATTCATCAC	
MT1G	NM_005950.1	F	CCCCAACTGCTCCTGTGCCG	202
		R	GGGAGCAGGGCTGTCCCGA	
MT1X	NM_005952.3	F	GCAAATGCAAAGAGTGCAAA	146
		R	ACAGCTGTCCTGGCATCAG	
NF_K_B1	NM_003998.2	F	CACGAATGACAGAGGCGTGTA	130
		R	TGGCGGATTAGCTCTTTTTCC	
RGL1	NM_015149.3	F	GCCGTCCCAAGGGACCGAATG	166
		R	GCCGCCTCTGGGTACGCTTC	
THBS1	NM_003246.2	F	CACGCTGCAGGACAGCAT	69
		R	GGCCGCCTCAGCTCATT	
TYMS	NM_001071.2	F	GGAGGAGTTGCTGTGGTTTATCAAG	108
		R	AGGCTGTCCAAAAAGTCTCGGG	
UNG	NM_003362.2	F	TTATGGTGAAACAGGGGAGA	176
		R	AGTGGAACTGGCAGAGACTG	

## Discussion

In an effort to characterize the response of colon cancer cells to HDACi, we analyzed the gene expression profile of two colon cancer cell lines following treatment with two HDACi, vorinostat and LBH589. Both HDACi resulted in significant inhibition of tumor cell proliferation, an accumulation of acetylated histones and the onset of apoptotic cell death. However, LBH589 exerted antiproliferative effects at significantly lower concentrations than vorinostat, consistent with previous reports utilizing these HDACi [25,34,41]. Specifically, the IC_50 _for LBH589 was in the single digit nanomolar range while vorinostat required concentrations in excess of 1 μM. The concentrations at which HDACi induce their antiproliferative effects is of importance particularly in the context of clinically achievable serum concentrations and the extrapolation of *in vitro *observations to clinical settings. Pharmacokinetic data from clinical trials following a twice-daily dose of vorinostat determined that the half-life was in the range of 1 - 3.5 h and maximal serum concentrations did not peak over 2 μM and rapidly diminished [27,42,43]. Of note, the half-life of LBH589 was determined to be in the order of 10-14 h and serum concentrations of 400 - 700 nM are achievable at doses which are well tolerated [28]. Therefore, the concentration of LBH589 required to achieve 50% growth inhibition in our colon cancer cells was well within clinically achievable concentrations whereas the concentration of vorinostat was within, but approaching the upper limit of reported serum concentration ranges.

The cDNA microarray analysis demonstrated that in each cell line that the gene expression profile was significantly altered after a 24 h exposure to either HDAC inhibitor, vorinostat (2 μM) or LBH589 (50 nM). Considering the mechanism of action of HDACi including histone acetylation-induced chromatin remodeling and the acetylation of non-histone proteins including transcription factors, it is intriguing that only 5-7% of genes in the colon cancer cell lines analyzed were modulated by HDACi treatment. However, our results are consistent with other microarray profiling experiments which reported as few as 2% and as high as 10% modulated by HDACi. These reports and the data presented herein would indicate that HDACi do not induce global gene expression changes and may instead target specific sets of genes. An important observation in this study was that vorinostat and LBH589 induced very similar transcriptional profiles within each cell line. As both of these agents are hydroxamate-class HDACi, this observation is somewhat expected. Additional studies have identified very similar transcriptional changes produced by the two hydroxamic-acid based HDACi, TSA and vorinostat, while also demonstrating a different gene expression profile obtained with the benzamide class HDACi MS-275 [14].

The analysis of our data demonstrates that HDACi induce significant cell-line specific effects on genes involved in the regulation of a number of critical tumor processes including angiogenesis, mitosis, DNA replication, recombination and repair and apoptosis. More specifically, the potent anti-angiogenic matrix glycoprotein thrombospondin-1 (THBS1), was significantly upregulated 14-fold in HCT116 cells at 24 h (Table 1). HT29 cells however, showed no modulation until 24 h post-treatment where only a modest increase of 2-fold was observed by qPCR. HDACi are reported to be potent inhibitors of tumor angiogenesis and induction of THBS1 has previously been reported following HDAC inhibition [44]. Similarly, in HCT116 the most heavily upregulated gene following treatment with either vorinostat or LBH589 was connective tissue growth factor (CTGF; Table 1). CTGF is a multifunctional secreted matricellular protein associated with fibrotic disorders, angiogenic regulation, and possibly tumor development [45]. Human tumors overexpressing CTGF demonstrated reduced microvessel density indicative of potential antiangiogenic properties, and ovarian tumors overexpressing CTGF demonstrated enhanced tumor cell invasion [46]. In HT29 cells, fibroblast growth factor 19 (FGF19) was significantly downregulated by both HDACi (Table 2). FGF19 binds to FGF receptor 4 and has been shown to mediate cell cycle progression, angiogenesis and promote tumor growth through the beta-catenin pathway. Knockdown of FGF19 in colon cancer cells decreased tumor growth *in vitro *and *in vivo *[47]. It is possible that the antiangiogenic and antitumor action of HDACi are mediated, in part, through modulation of key angiogenic regulators such as these and would indicate that HDACi may potentiate the therapeutic efficacy when used in combination with inhibitors of tumor angiogenesis.

In HT29 cells, microarray analysis identified that both HDACi induce a potent downregulation of the anti-apoptotic caspase inhibitor protein AVEN. qPCR confirmed that AVEN is significantly downregulated in HT29 cells by vorinostat and LBH589 >5-fold at 24 h and only modestly regulated in HCT116 cells <2-fold at 24 h. AVEN is reported to inhibit caspase activation through inhibition of APAF-1 self-association [48]. The downregulation of AVEN would suggest that HDACi-induced apoptosis in the HT29 cells may be regulated in part via the mitochondria, supporting the mechanism of oxidative stress injury as previously reported [18].

We also observed significant cell-line-specific alterations in genes involved in mitosis. Aurora kinase B was identified as downregulated by both vorinostat and LBH589 in HCT116 cells. The Aurora kinase family are critical regulators of mitotic cell division having roles in centrosome function, mitotic spindle formation, chromosome segregation and cytokinesis [49]. Overexpression of Aurora kinases A and B have been linked to genetic instability and are frequently overexpressed in solid tumors such as colorectal cancer [50,51] and inhibition of aurora kinases has become an attractive therapeutic strategy with multiple inhibitors in clinical development. Of note, recent studies have reported that LBH589 induces the degradation of aurora kinase A and B in renal and non-small cell lung cancer cells resulting in G2/M arrest and apoptosis [52,53]. Interestingly, we observed downregulation of aurora kinase B with HDACi treatment only in the HCT116 cells where a potent G2/M arrest and significant apoptosis was observed (Table 1, Figure 5B).

Approximately 18% of the DEGs identified after HDACi treatment were modulated in a similar manner in both cell lines. This core set of genes encompass genes involved in cell cycle, nucleotide metabolism, nucleosome assembly and apoptosis. We identified a panel of 11 genes, 6 up- and 5 downregulated by both HDACi in both cell lines. Previously, Glaser *et al*. identified a core set of 13 genes regulated by three HDACis in bladder and breast cancer carcinoma cell line models. Upon comparison, one upregulated gene (histone H2B) and one down-regulated gene (thymidylate synthase) are consistent between our core gene set and those reported by Glaser *et al*. [14]. One of the primary reasons for this is that out core gene set was defined solely from colon cancer cells which are physiologically distinct from both bladder and breast cancers and may employ different mechanisms of gene expression regulation. An additional study analyzed the effects of HDACi in renal cancer cells and identified consistent directional modulation of short-chain alcohol dehydrogenase, aldo-keto reductase and fibroblast growth factor gene families [54].

Two genes within our core set of HDACi-modulated genes are directly involved in nucleotide metabolism and DNA repair. Downregulation of both thymidylate synthase (TYMS) and UNG was observed in both cell lines following treatment with either HDACi. Thymidylate synthase is essential for the *de novo *synthesis of thymidylate, an essential precursor required for DNA replication and repair. UNG is the gene encoding uracil-DNA glycosylase, a base excision repair protein involved in uracil excision from DNA. Both these enzymes are reported to mediate response to the antimetabolite class of chemotherapeutic agents including inhibitors of TS such as 5-fluorouracil [55,56]. A number of other studies have confirmed that downregulation of TS mRNA and protein is a common event in response to HDACi treatment [14,25,57]. We recently confirmed that downregulation of TS was a common event in an extended panel of colon cell lines and was driven primarily through a transcriptional mechanism in response to HDAC inhibition. This interaction resulted in synergistic antiproliferative effects between HDACi and 5-FU in colon cancer cells [25] supporting the concept that HDACi-mediated alterations in known drug targets may provide opportunity for new therapeutic combinations.

Short-chain alcohol dehydrogenase family member 2 (DHRS2) was identified as the most heavily induced gene by HDACi in our core set of genes. DHRS2 was originally identified following its upregulation by treatment with butyrate and was later confirmed to be involved in the differentiation of monocytes to dendritic cells [58,59]. HDACi treatment is reported to induce cellular differentiation and induction of pro-differentiation genes such as DHRS2 is a plausible mechanism [60].

MT1X and MT1G were both heavily induced in both cell lines by HDACi treatment. These genes encode two highly inducible ubiquitous proteins belonging to a family of cysteine-rich metallothionein proteins. Metallothioneins can bind to both physiological and xenobiotic heavy metals [61]. Previous studies have identified regulation of other metallothionein family members in response to HDACi [14]. MT1G is reported to be a tumor suppressor gene and is frequently epigenetically silenced in a number of human malignancies [62,63]. Although the mechanism that results in the induction of metallothionein proteins is unknown, both the MT1X and MT1G genes map to chromosome 16q13 and it is likely that HDACi-mediated events in this region such as chromatin relaxation result in the increased transcription of both of these genes.

NF-κB regulates the expression of a significant number of genes involved in immune response, angiogenesis, cell adhesion, proliferation, differentiation, and apoptosis [64,65]. The NFKB1 gene encodes the predominant p50/p105 form and represents one of the core genes significantly downregulated by HDACi treatment in this study. As such, many different types of human tumors have dysregulated NF-κB, primarily via constitutive activation that mediates continued cell proliferation and averts the onset of apoptosis [66]. Downregulation of NF-κB is a likely mechanism by which HDACi induce aspects of their apoptotic effects in colon cancer cells. We also identified the IL-1 receptor associated kinase (IRAK1) as consistently downregulated by HDACi in our core set of genes. IRAK1 encodes the interleukin-1 receptor-associated kinase 1 which is reported to be partially responsible for IL1-induced upregulation of NF-κB [67] and was one of ~100 genes identified as consistently upregulated in a microarray meta-comparison of genes upregulated in solid tumors of epithelial origin [68].

Our core set of genes includes the histone family member HIST1H2BD which encodes the histone H2B protein and was >3-fold induced by HDACi treatment. HIST1H2BD has previously been reported to be induced by HDACi treatment [14]. While the mechanism of induction of this gene is unknown, it is located within the large histone gene cluster on chromosome 6p22-p21.3 and it is likely that the HDACi-induced alterations in this region, possibly through chromatin relaxation allowing transcriptional machinery access, results in this induction.

We have analyzed the gene expression profiles of two of the most clinically advanced hydroxamate class HDACi, vorinostat and LBH589, in two colon cancer cell line models. We identified significant overlap in differentially expressed gene profiles for vorinostat and LBH589 within each cell line indicating similar mechanism of action for these HDACi. Interestingly, we also identified a strong cell-line dependence of gene expression changes induced by these HDACi with only 18% commonality in HDACi-induced DEGs. Within this gene expression overlap, we identified a core set of 6 up- and 5 downregulated genes that are regulated by both of HDACi in both cell lines. Defining a core set of genes that represent markers of HDAC inhibition is an important first step in the identification and validation of clinical markers for evaluating HDACi target inhibition and efficacy. Currently, analysis of histone acetylation from tumor tissue and more frequently from isolated peripheral blood mononuclear cells is used as evidence of HDACi biological activity. However, histone acetylation following HDACi treatment has been shown to be highly reversible and often inconsistent. A panel of HDACi-regulated genes may provide a more sensitive and reliable means to determining the efficacy of HDACi treatment in the clinic. We also identified alterations in additional pathways which may enhance the therapeutic potential of both conventional and targeted therapeutics, including genes involved in angiogenesis, nucleotide metabolism and mitosis. As HDACi advance in clinical development, these agents are likely to be incorporated into combination treatment strategies with both conventional and novel chemotherapeutic agents. Therefore, the identification of pathways and drug targets modulated by HDAC inhibition could be critically important in elucidating their disease-specific mechanism of action and assisting in the identification of effective drug combination partners.

## Conclusion

This study identified HDACi-induced alterations in critical genes involved in nucleotide metabolism, angiogenesis, mitosis and cell survival which may represent potential intervention points for novel therapeutic combinations in colon cancer. This information will assist in the identification of novel pathways and targets that are modulated by HDACi, providing much-needed information on HDACi mechanism of action and providing rationale for novel drug combination partners. We identified a core signature of 11 genes which were modulated by both vorinostat and LBH589 in a similar manner in both cell lines. These core genes will assist in the development and validation of a common gene set which may represent a molecular signature of HDAC inhibition in colon cancer.

## Abbreviations

HDAC: histone deacetylase; HDACi: histone deacetylase inhibitor; HAT: histone acetyl transferase; FDR: false discovery rate; CTCL: cutaneous T-cell lymphoma; metallothionein; MT: thymidylate synthase (gene); TYMS: (protein) TS; NFkB: nuclear factor kappa-light-chain-enhancer of activated B cells; qPCR: quantitative reverse-transcriptase PCR; DEG(s): differentially expressed gene(s).

## Competing interests

HJL has received financial support for consultation and clinical trial support from Novartis and clinical trial support from Merck. The remaining authors of this manuscript have no competing interests to declare.

## Authors' contributions

All authors conceived the experiment design. MJL, WF and PMW performed the cell culture, RNA extraction and quality control prior to microarray analysis. MJL, PMW and WF performed the quantitative RT-PCR. MJL and PMW performed the growth inhibition analysis, Western blotting and flow cytometric analysis. SG supervised all statistical analyses. All authors interpreted the results. The manuscript was drafted by MJL, PMW and WF. All authors approved the final manuscript.

## Pre-publication history

The pre-publication history for this paper can be accessed here:

http://www.biomedcentral.com/1755-8794/2/67/prepub

## References

[B1] Struhl K Histone acetylation and transcriptional regulatory mechanisms Genes Dev 1998 12 5 599 606 10.1101/gad.12.5.599 9499396

[B2] Glaser KB HDAC inhibitors: clinical update and mechanism-based potential Biochem Pharmacol 2007 74 5 659 671 10.1016/j.bcp.2007.04.007 17498667

[B3] Bali P Pranpat M Bradner J Balasis M Fiskus W Guo F Rocha K Kumaraswamy S Boyapalle S Atadja P Inhibition of histone deacetylase 6 acetylates and disrupts the chaperone function of heat shock protein 90: a novel basis for antileukemia activity of histone deacetylase inhibitors J Biol Chem 2005 280 29 26729 26734 10.1074/jbc.C500186200 15937340

[B4] Glozak MA Sengupta N Zhang X Seto E Acetylation and deacetylation of non-histone proteins Gene 2005 363 15 23 10.1016/j.gene.2005.09.010 16289629

[B5] Hubbert C Guardiola A Shao R Kawaguchi Y Ito A Nixon A Yoshida M Wang XF Yao TP HDAC6 is a microtubule-associated deacetylase Nature 2002 417 6887 455 458 10.1038/417455a 12024216

[B6] Prystowsky MB Adomako A Smith RV Kawachi N McKimpson W Atadja P Chen Q Schlecht NF Parish JL Childs G The histone deacetylase inhibitor LBH589 inhibits expression of mitotic genes causing G2/M arrest and cell death in head and neck squamous cell carcinoma cell lines J Pathol 2009 218 4 467 77 10.1002/path.2554 19402126

[B7] Stevens FE Beamish H Warrener R Gabrielli B Histone deacetylase inhibitors induce mitotic slippage Oncogene 2008 27 10 1345 1354 10.1038/sj.onc.1210779 17828304

[B8] Ishii S Kurasawa Y Wong J Yu-Lee LY Histone deacetylase 3 localizes to the mitotic spindle and is required for kinetochore-microtubule attachment Proc Natl Acad Sci USA 2008 105 11 4179 4184 10.1073/pnas.0710140105 18326024 PMC2393771

[B9] Marquard L Gjerdrum LM Christensen IJ Jensen PB Sehested M Ralfkiaer E Prognostic significance of the therapeutic targets histone deacetylase 1, 2, 6 and acetylated histone H4 in cutaneous T-cell lymphoma Histopathology 2008 53 3 267 277 10.1111/j.1365-2559.2008.03109.x 18671804 PMC2675007

[B10] Abbas A Gupta S The role of histone deacetylases in prostate cancer Epigenetics 2008 3 6 300 309 19029799 10.4161/epi.3.6.7273 PMC2683066

[B11] Suzuki J Chen YY Scott GK Devries S Chin K Benz CC Waldman FM Hwang ES Protein acetylation and histone deacetylase expression associated with malignant breast cancer progression Clin Cancer Res 2009 15 9 3163 3171 10.1158/1078-0432.CCR-08-2319 19383825 PMC3746548

[B12] Hanigan CL Van Engeland M De Bruine AP Wouters KA Weijenberg MP Eshleman JR Herman JG An inactivating mutation in HDAC2 leads to dysregulation of apoptosis mediated by APAF1 Gastroenterology 2008 135 5 1654 1664 10.1053/j.gastro.2008.07.078 18834886

[B13] Wilson AJ Byun DS Nasser S Murray LB Ayyanar K Arango D Figueroa M Melnick A Kao GD Augenlicht LH HDAC4 promotes growth of colon cancer cells via repression of p21 Mol Biol Cell 2008 19 10 4062 4075 10.1091/mbc.E08-02-0139 18632985 PMC2555950

[B14] Glaser KB Staver MJ Waring JF Stender J Ulrich RG Davidsen SK Gene expression profiling of multiple histone deacetylase (HDAC) inhibitors: defining a common gene set produced by HDAC inhibition in T24 and MDA carcinoma cell lines Mol Cancer Ther 2003 2 2 151 163 12589032

[B15] Bolden JE Peart MJ Johnstone RW Anticancer activities of histone deacetylase inhibitors Nat Rev Drug Discov 2006 5 9 769 784 10.1038/nrd2133 16955068

[B16] Marks P Rifkind RA Richon VM Breslow R Miller T Kelly WK Histone deacetylases and cancer: causes and therapies Nat Rev Cancer 2001 1 3 194 202 10.1038/35106079 11902574

[B17] Drummond DC Noble CO Kirpotin DB Guo Z Scott GK Benz CC Clinical development of histone deacetylase inhibitors as anticancer agents Annu Rev Pharmacol Toxicol 2005 45 495 528 10.1146/annurev.pharmtox.45.120403.095825 15822187

[B18] Portanova P Russo T Pellerito O Calvaruso G Giuliano M Vento R Tesoriere G The role of oxidative stress in apoptosis induced by the histone deacetylase inhibitor suberoylanilide hydroxamic acid in human colon adenocarcinoma HT-29 cells Int J Oncol 2008 33 2 325 331 18636153

[B19] Richon VM Emiliani S Verdin E Webb Y Breslow R Rifkind RA Marks PA A class of hybrid polar inducers of transformed cell differentiation inhibits histone deacetylases Proc Natl Acad Sci USA 1998 95 6 3003 3007 10.1073/pnas.95.6.3003 9501205 PMC19684

[B20] Richon VM Garcia-Vargas J Hardwick JS Development of vorinostat: Current applications and future perspectives for cancer therapy Cancer Lett 2009 280 2 201 210 10.1016/j.canlet.2009.01.002 19181442

[B21] Atadja P Development of the pan-DAC inhibitor panobinostat (LBH589): Successes and challenges Cancer Lett 2009 280 2 233 41 10.1016/j.canlet.2009.02.019 19344997

[B22] American Cancer Society Cancer Facts & Figures 2008 2008 Atlanta: American Cancer Society http://www.cancer.org

[B23] Douillard JY Cunningham D Roth AD Navarro M James RD Karasek P Jandik P Iveson T Carmichael J Alakl M Irinotecan combined with fluorouracil compared with fluorouracil alone as first-line treatment for metastatic colorectal cancer: a multicentre randomised trial Lancet 2000 355 9209 1041 1047 10.1016/S0140-6736(00)02034-1 10744089

[B24] Giacchetti S Perpoint B Zidani R Le Bail N Faggiuolo R Focan C Chollet P Llory JF Letourneau Y Coudert B Phase III multicenter randomized trial of oxaliplatin added to chronomodulated fluorouracil-leucovorin as first-line treatment of metastatic colorectal cancer J Clin Oncol 2000 18 1 136 147 10623704 10.1200/JCO.2000.18.1.136

[B25] Fazzone W Wilson PM Labonte MJ Lenz HJ Ladner RD Histone deacetylase inhibitors suppress thymidylate synthase gene expression and synergize with the fluoropyrimidines in colon cancer cells Int J Cancer 2009 125 2 463 73 10.1002/ijc.24403 19384949

[B26] Richon VM Sandhoff TW Rifkind RA Marks PA Histone deacetylase inhibitor selectively induces p21WAF1 expression and gene-associated histone acetylation Proc Natl Acad Sci USA 2000 97 18 10014 10019 10.1073/pnas.180316197 10954755 PMC27656

[B27] Kelly WK Richon VM O'Connor O Curley T MacGregor-Curtelli B Tong W Klang M Schwartz L Richardson S Rosa E Phase I clinical trial of histone deacetylase inhibitor: suberoylanilide hydroxamic acid administered intravenously Clin Cancer Res 2003 9 10 Pt 1 3578 3588 14506144

[B28] Giles F Fischer T Cortes J Garcia-Manero G Beck J Ravandi F Masson E Rae P Laird G Sharma S A phase I study of intravenous LBH589, a novel cinnamic hydroxamic acid analogue histone deacetylase inhibitor, in patients with refractory hematologic malignancies Clin Cancer Res 2006 12 15 4628 4635 10.1158/1078-0432.CCR-06-0511 16899611

[B29] Tumber A Collins LS Petersen KD Thougaard A Christiansen SJ Dejligbjerg M Jensen PB Sehested M Ritchie JW The histone deacetylase inhibitor PXD101 synergises with 5-fluorouracil to inhibit colon cancer cell growth in vitro and in vivo Cancer Chemother Pharmacol 2007 60 2 275 283 10.1007/s00280-006-0374-7 17124594

[B30] Zhang QL Wang L Zhang YW Jiang XX Yang F Wu WL Janin A Chen Z Shen ZX Chen SJ The proteasome inhibitor bortezomib interacts synergistically with the histone deacetylase inhibitor suberoylanilide hydroxamic acid to induce T-leukemia/lymphoma cells apoptosis Leukemia 2009 23 8 1507 14 10.1038/leu.2009.41 19282831

[B31] Pitts TM Morrow M Kaufman SA Tentler JJ Eckhardt SG Vorinostat and bortezomib exert synergistic antiproliferative and proapoptotic effects in colon cancer cell models Mol Cancer Ther 2009 8 2 342 349 10.1158/1535-7163.MCT-08-0534 19174560 PMC2813767

[B32] Frew AJ Lindemann RK Martin BP Clarke CJ Sharkey J Anthony DA Banks KM Haynes NM Gangatirkar P Stanley K Combination therapy of established cancer using a histone deacetylase inhibitor and a TRAIL receptor agonist Proc Natl Acad Sci USA 2008 105 32 11317 11322 10.1073/pnas.0801868105 18685088 PMC2516269

[B33] Zhang W Peyton M Xie Y Soh J Minna JD Gazdar AF Frenkel EP Histone deacetylase inhibitor romidepsin enhances anti-tumor effect of erlotinib in non-small cell lung cancer (NSCLC) cell lines J Thorac Oncol 2009 4 2 161 166 10.1097/JTO.0b013e318194fae7 19179890 PMC2758160

[B34] Edwards A Li J Atadja P Bhalla K Haura EB Effect of the histone deacetylase inhibitor LBH589 against epidermal growth factor receptor-dependent human lung cancer cells Mol Cancer Ther 2007 6 9 2515 2524 10.1158/1535-7163.MCT-06-0761 17876048

[B35] Wilson PM Fazzone W LaBonte MJ Deng J Neamati N Ladner RD Novel opportunities for thymidylate metabolism as a therapeutic target Mol Cancer Ther 2008 7 9 3029 3037 10.1158/1535-7163.MCT-08-0280 18790783 PMC2597111

[B36] Wilson PM Fazzone W Labonte MJ Lenz HJ Ladner RD Regulation of human dUTPase gene expression and p53-mediated transcriptional repression in response to oxaliplatin-induced DNA damage Nucleic Acids Res 2008 37 1 78 95 10.1093/nar/gkn910 19015155 PMC2615606

[B37] Bolstad BM Irizarry RA Astrand M Speed TP A comparison of normalization methods for high density oligonucleotide array data based on variance and bias Bioinformatics 2003 19 2 185 193 10.1093/bioinformatics/19.2.185 12538238

[B38] Benjamini Y Hochberg Y Controlling the false discovery rate: a practical and powerful approach to multiple testing J R Statist Soc 1995 57 1 289 300

[B39] Livak KJ Schmittgen TD Analysis of relative gene expression data using real-time quantitative PCR and the 2(-Delta Delta C(T)) Method Methods 2001 25 4 402 408 10.1006/meth.2001.1262 11846609

[B40] Beckman KB Lee KY Golden T Melov S Gene expression profiling in mitochondrial disease: assessment of microarray accuracy by high-throughput Q-PCR Mitochondrion 2004 4 5-6 453 470 10.1016/j.mito.2004.07.029 16120406

[B41] George P Bali P Annavarapu S Scuto A Fiskus W Guo F Sigua C Sondarva G Moscinski L Atadja P Combination of the histone deacetylase inhibitor LBH589 and the hsp90 inhibitor 17-AAG is highly active against human CML-BC cells and AML cells with activating mutation of FLT-3 Blood 2005 105 4 1768 1776 10.1182/blood-2004-09-3413 15514006

[B42] Kelly WK O'Connor OA Krug LM Chiao JH Heaney M Curley T MacGregore-Cortelli B Tong W Secrist JP Schwartz L Phase I study of an oral histone deacetylase inhibitor, suberoylanilide hydroxamic acid, in patients with advanced cancer J Clin Oncol 2005 23 17 3923 3931 10.1200/JCO.2005.14.167 15897550 PMC1855284

[B43] Fakih MG Pendyala L Fetterly G Toth K Zwiebel JA Espinoza-Delgado I Litwin A Rustum YM Ross ME Holleran JL A phase I, pharmacokinetic and pharmacodynamic study on vorinostat in combination with 5-fluorouracil, leucovorin, and oxaliplatin in patients with refractory colorectal cancer Clin Cancer Res 2009 15 9 3189 3195 10.1158/1078-0432.CCR-08-2999 19383814 PMC4134938

[B44] Kang JH Kim MJ Chang SY Sim SS Kim MS Jo YH CCAAT box is required for the induction of human thrombospondin-1 gene by trichostatin A J Cell Biochem 2008 104 4 1192 1203 10.1002/jcb.21697 18275041

[B45] Cicha I Goppelt-Struebe M Connective tissue growth factor: Context-dependent functions and mechanisms of regulation Biofactors 2009 35 2 200 208 10.1002/biof.30 19449449

[B46] Barbolina MV Adley BP Kelly DL Shepard J Fought AJ Scholtens D Penzes P Shea LD Stack MS Downregulation of connective tissue growth factor by three-dimensional matrix enhances ovarian carcinoma cell invasion Int J Cancer 2009 125 4 816 25 10.1002/ijc.24347 19382180 PMC2849282

[B47] Pai R Dunlap D Qing J Mohtashemi I Hotzel K French DM Inhibition of fibroblast growth factor 19 reduces tumor growth by modulating beta-catenin signaling Cancer Res 2008 68 13 5086 5095 10.1158/0008-5472.CAN-07-2325 18593907

[B48] Chau BN Cheng EH Kerr DA Hardwick JM Aven, a novel inhibitor of caspase activation, binds Bcl-xL and Apaf-1 Mol Cell 2000 6 1 31 40 10.1016/S1097-2765(00)00005-8 10949025

[B49] Carmena M Earnshaw WC The cellular geography of aurora kinases Nat Rev Mol Cell Biol 2003 4 11 842 854 10.1038/nrm1245 14625535

[B50] Bischoff JR Anderson L Zhu Y Mossie K Ng L Souza B Schryver B Flanagan P Clairvoyant F Ginther C A homologue of Drosophila aurora kinase is oncogenic and amplified in human colorectal cancers Embo J 1998 17 11 3052 3065 10.1093/emboj/17.11.3052 9606188 PMC1170645

[B51] Katayama H Ota T Jisaki F Ueda Y Tanaka T Odashima S Suzuki F Terada Y Tatsuka M Mitotic kinase expression and colorectal cancer progression J Natl Cancer Inst 1999 91 13 1160 1162 10.1093/jnci/91.13.1160 10393726

[B52] Cha TL Chuang MJ Wu ST Sun GH Chang SY Yu DS Huang SM Huan SK Cheng TC Chen TT Dual degradation of aurora A and B kinases by the histone deacetylase inhibitor LBH589 induces G2-M arrest and apoptosis of renal cancer cells Clin Cancer Res 2009 15 3 840 850 10.1158/1078-0432.CCR-08-1918 19188154

[B53] Zhang XH Rao M Loprieato JA Hong JA Zhao M Chen GZ Humphries AE Nguyen DM Trepel JB Yu X Aurora A, Aurora B and survivin are novel targets of transcriptional regulation by histone deacetylase inhibitors in non-small cell lung cancer Cancer Biol Ther 2008 7 9 1388 1397 18708766 10.4161/cbt.7.9.6415

[B54] Tavares TS Nanus D Yang XJ Gudas LJ Gene microarray analysis of human renal cell carcinoma: the effects of HDAC inhibition and retinoid treatment Cancer Biol Ther 2008 7 10 1607 1618 18769122 10.4161/cbt.7.10.6584 PMC3060607

[B55] Popat S Matakidou A Houlston RS Thymidylate synthase expression and prognosis in colorectal cancer: a systematic review and meta-analysis J Clin Oncol 2004 22 3 529 536 10.1200/JCO.2004.05.064 14752076

[B56] Dusseau C Murray GI Keenan RA O'Kelly T Krokan HE McLeod HL Analysis of uracil DNA glycosylase in human colorectal cancer Int J Oncol 2001 18 2 393 399 11172609 10.3892/ijo.18.2.393

[B57] Lee JH Park JH Jung Y Kim JH Jong HS Kim TY Bang YJ Histone deacetylase inhibitor enhances 5-fluorouracil cytotoxicity by down-regulating thymidylate synthase in human cancer cells Mol Cancer Ther 2006 5 12 3085 3095 10.1158/1535-7163.MCT-06-0419 17172411

[B58] Gabrielli F Donadel G Bensi G Heguy A Melli M A nuclear protein, synthesized in growth-arrested human hepatoblastoma cells, is a novel member of the short-chain alcohol dehydrogenase family Eur J Biochem 1995 232 2 473 477 10.1111/j.1432-1033.1995.473zz.x 7556196

[B59] Donadel G Garzelli C Frank R Gabrielli F Identification of a novel nuclear protein synthesized in growth-arrested human hepatoblastoma HepG2 cells Eur J Biochem 1991 195 3 723 729 10.1111/j.1432-1033.1991.tb15759.x 1847869

[B60] Carew JS Giles FJ Nawrocki ST Histone deacetylase inhibitors: mechanisms of cell death and promise in combination cancer therapy Cancer Lett 2008 269 1 7 17 10.1016/j.canlet.2008.03.037 18462867

[B61] Thirumoorthy N Manisenthil Kumar KT Shyam Sundar A Panayappan L Chatterjee M Metallothionein: an overview World J Gastroenterol 2007 13 7 993 996 17373731 10.3748/wjg.v13.i7.993 PMC4146885

[B62] Ferrario C Lavagni P Gariboldi M Miranda C Losa M Cleris L Formelli F Pilotti S Pierotti MA Greco A Metallothionein 1G acts as an oncosupressor in papillary thyroid carcinoma Lab Invest 2008 88 5 474 481 10.1038/labinvest.2008.17 18332874

[B63] Henrique R Jeronimo C Hoque MO Nomoto S Carvalho AL Costa VL Oliveira J Teixeira MR Lopes C Sidransky D MT1G hypermethylation is associated with higher tumor stage in prostate cancer Cancer Epidemiol Biomarkers Prev 2005 14 5 1274 1278 10.1158/1055-9965.EPI-04-0659 15894685

[B64] Terragni J Graham JR Adams KW Schaffer ME Tullai JW Cooper GM Phosphatidylinositol 3-kinase signaling in proliferating cells maintains an anti-apoptotic transcriptional program mediated by inhibition of FOXO and non-canonical activation of NFkappaB transcription factors BMC Cell Biol 2008 9 6 10.1186/1471-2121-9-6 18226221 PMC2268685

[B65] Tergaonkar V NFkappaB pathway: a good signaling paradigm and therapeutic target Int J Biochem Cell Biol 2006 38 10 1647 1653 10.1016/j.biocel.2006.03.023 16766221

[B66] Bernal-Mizrachi L Lovly CM Ratner L The role of NF-{kappa}B-1 and NF-{kappa}B-2-mediated resistance to apoptosis in lymphomas Proc Natl Acad Sci USA 2006 103 24 9220 9225 10.1073/pnas.0507809103 16751281 PMC1482593

[B67] Vig E Green M Liu Y Donner DB Mukaida N Goebl MG Harrington MA Modulation of tumor necrosis factor and interleukin-1-dependent NF-kappaB activity by mPLK/IRAK J Biol Chem 1999 274 19 13077 13084 10.1074/jbc.274.19.13077 10224059

[B68] Pilarsky C Wenzig M Specht T Saeger HD Grutzmann R Identification and validation of commonly overexpressed genes in solid tumors by comparison of microarray data Neoplasia 2004 6 6 744 750 10.1593/neo.04277 15720800 PMC1531678

